# Supplementary material for: Functions of COP1/SPA E3 Ubiquitin Ligase Mediated by MpCRY in the Liverwort Marchantia polymorpha under Blue Light
Source: Int J Mol Sci. 2021 Dec 23;23(1):158. doi: 10.3390/ijms23010158 (PMC8745113; doi:10.3390/ijms23010158)
Supplement: Supplementary file 1 [file ijms-23-00158-s001.zip › ijms-1504820-suppl/supplementary figures.pdf]

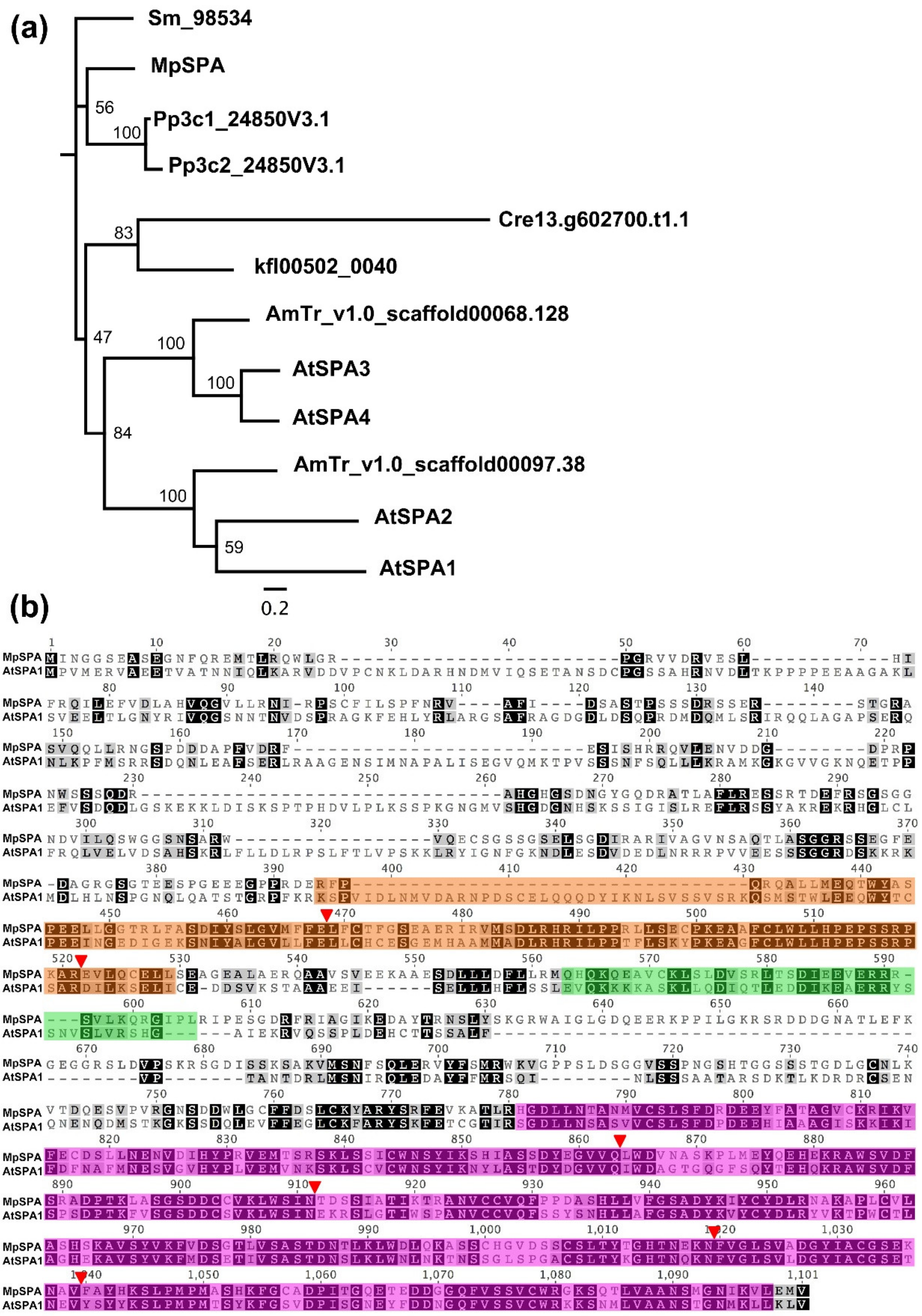

Figure S1. Phylogenetic analysis of SPA proteins from green algae and land plants. (a) Phylogenetic relationships of plant SPA proteins. The tree was constructed using 12 SPA proteins from various plant species. Posterior probabilities are indicated at the nodes. At, *Arabidopsis thaliana*; AmTr, *Amborella trichopoda*; Sm, *Selaginella moellendorffii*; Pp, Physcomitrium patens; Kfl, Klebsormidium nitens; Cre, Chlamydomonas reinhardtii. Bar indicates 0.2 substitutions per site. **(b)** Alignment of amino acid sequences of the SPA protein from *M. polymorpha* and *A.*

thaliana. Identical and similar amino acid residues are highlighted with black and gray boxes, respectively. Orange background sequences indicate the kinase domain. Green background sequences indicate the coiled-coil domain. Purple background sequences indicate the WD40 domain. Red arrowheads indicate positions corresponding to intron insertion in the genes.

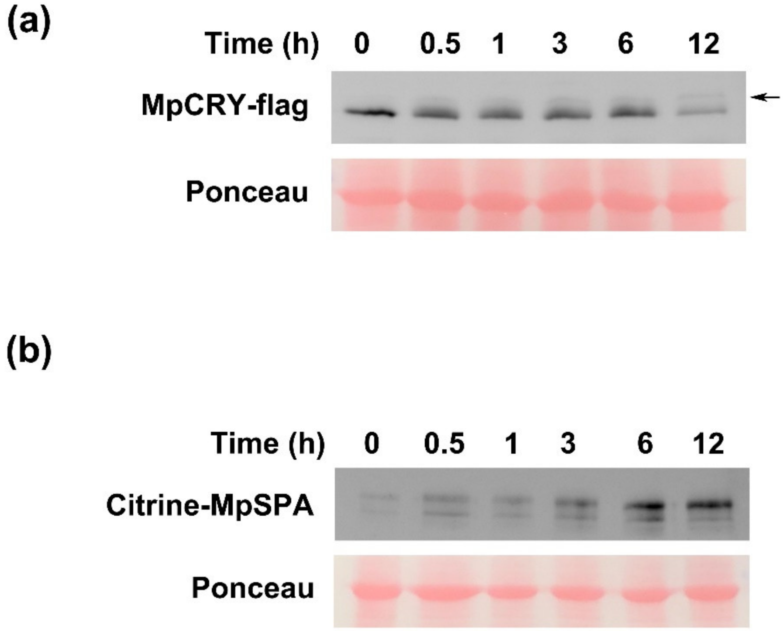

Figure S2. Levels of Citrine-MpSPA1 and MpCRY-Flag proteins in *M. polymorpha* under BL. **(a)** Gemmae expressing MpCRY-Flag were plated on Gamborg's B5 medium under white light for 12 days and then were treated in the dark for 2 days. The gemmalings were then transferred to BL (30 μmol m^{-2} s^{-1}) for indicated time. The levels of MpCRY-Flag were estimated by immunoblot. The immunoprecipitation signals were probed by anti-Flag. Black arrow represents phosphorylated MpCRY-Flag. **(b)** Gemmae expressing Citrine-MpSPA were plated on Gamborg's B5 medium under white light for 12 days and then were treated in the dark for 2 days. The gemmalings were then transferred to BL (30 μmol m^{-2} s^{-1}) for indicated time. The levels of Citrine-MpSPA were estimated by immunoblot. The immunoprecipitation signals were probed by anti-Citrine.

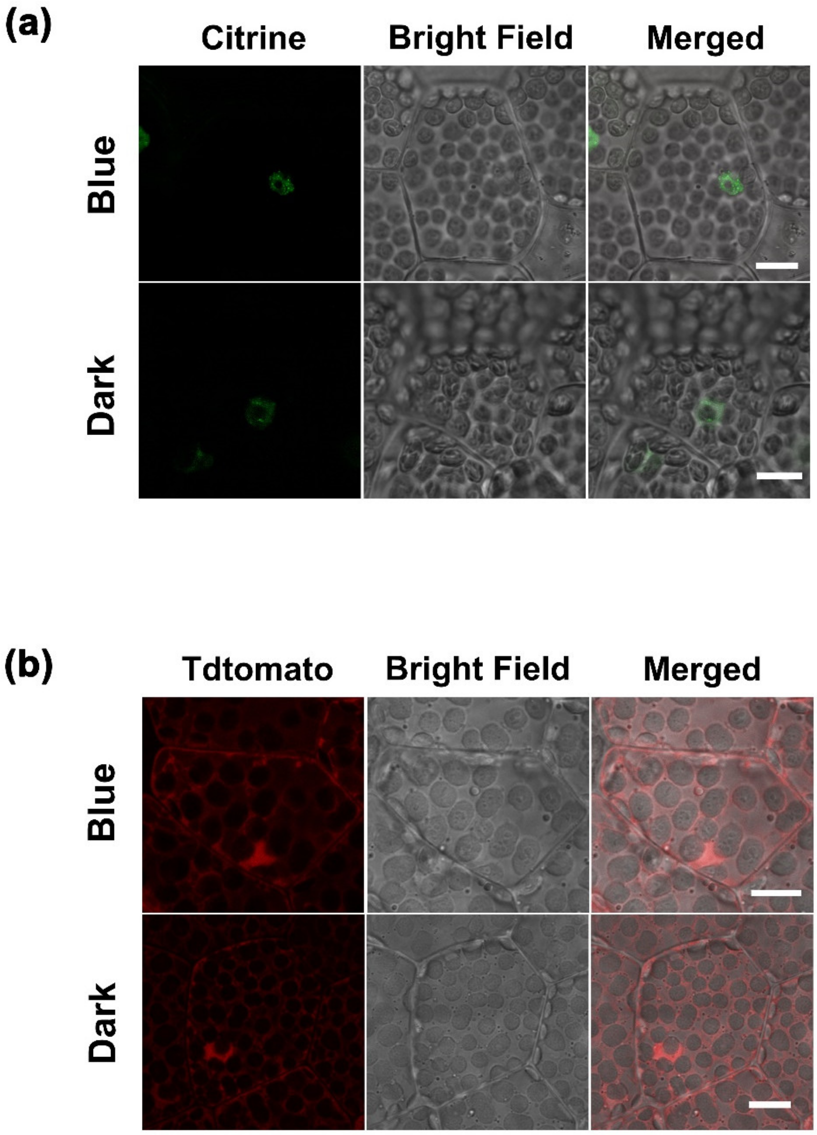

Figure S3. The subcellular localization of MpCRY-Tdtomato and Citrine-MpSPA1 in *M. polymorpha*. (A) Gemmae expressing MpCRY-Tdtomato were plated on Gamborg's B5 medium and imbibed in the dark for 3 days. Then the gemmalings were incubated under BL (30 μmol m^{-2} s^{-1}) or kept in the dark for 6 h. Leica TCS SP8X confocal microscope was used to observe the gemmalings. Bar = 10 μm. **(B)** Gemmae expressing Citrine-MpSPA were plated on Gamborg's B5 medium and imbibed in the dark for 3 days. Then the gemmalings were incubated under BL (30 μmol m^{-2} s^{-1}) or kept in the dark for 6 h. Leica TCS SP8X confocal microscope was used to observe the gemmalings. Bar = 10 μm.

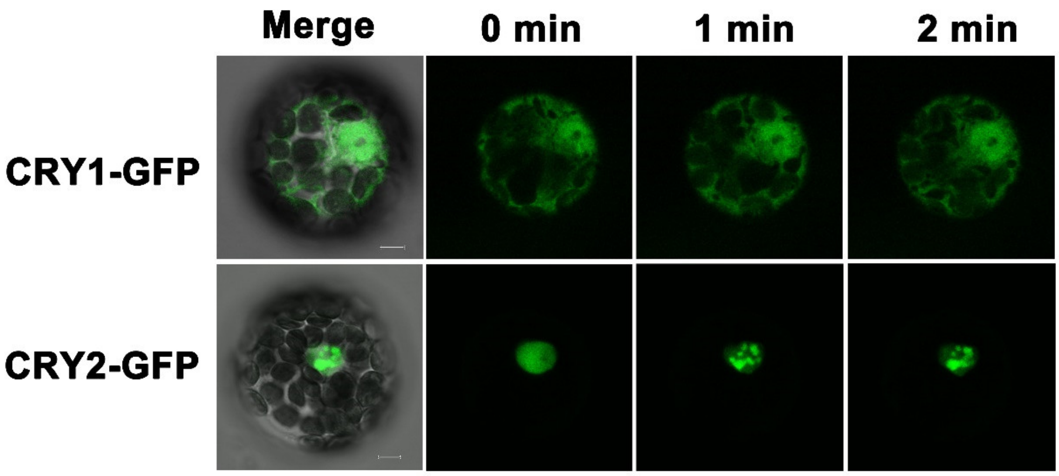

Figure S4. AtCRY1 failed to form photobodies when expressed alone. Plasmids expressing CRY1-GFP and CRY2-GFP were transfected into *Arabidopsis* protoplasts. After 14h, dark incubated protoplasts were activated by blue light (488 nm) laser at indicated time. scale bar = 5 μm. Similar results were observed in 3 independent repeats.
